# Media attention toward COVID-19 across 18 countries: The influence of cultural values and pandemic severity

**DOI:** 10.1371/journal.pone.0271961

**Published:** 2022-12-07

**Authors:** Reuben Ng, Yi Wen Tan

**Affiliations:** 1 Lee Kuan Yew School of Public Policy, National University of Singapore, Singapore, Singapore; 2 Lloyd’s Register Foundation Institute for the Public Understanding of Risk, National University of Singapore, Singapore, Singapore; Johannes Gutenberg Universitat Mainz, GERMANY

## Abstract

**Background:**

Current media studies of COVID-19 devote asymmetrical attention to social media, in contrast, newspapers have received comparatively less attention. Newspapers are an integral source of current information—that are syndicated and amplified by social media to a wide global audience. This is the first-known study to investigate the impact of cultural values and pandemic severity on media attention towards COVID-19. Findings lay the groundwork for targeted public health communications that are culturally nuanced.

**Objective:**

We investigated the impact of cultural values and pandemic severity on Media Attention towards COVID-19 across 18 countries.

**Methods:**

We tracked the global volume of COVID-19 coverage (to measure media attention) over 8 months in a news media database of 12 billion words with 30 million articles from over 7,000 news media sites. Predictors of Media Attention towards COVID-19 came from the Oxford COVID-19 Government Response Tracker (incidence and mortality) and Hofstede’s Cultural Values.

**Results:**

Media attention toward COVID-19 increased 55 times over 8 months. Higher rates of new cases and deaths predicted this exponential increase. Countries with higher power distance, uncertainty avoidance, and long-term orientation, were associated with increased media attention, controlling for covariates.

**Conclusions:**

Cultural values play a significant role in the news media’s attention toward COVID-19, controlling for pandemic severity. We provided a framework to design targeted public health communications that are culturally nuanced.

## Introduction

The COVID-19 outbreak has taken a toll across multiple aspects of daily lives. Current media studies of COVID-19 devote asymmetrical attention on social media [1–3], in contrast, newspapers have received comparatively less attention. Newspapers are an integral source of current information—that are syndicated and amplified by social media to a wide global audience. There is ample research concerning the use of social media like Twitter during the pandemic, but only a handful have considered the cultural impact on online news media as part of their investigations [4]. There is virtually no study that examines cultural values that underly the volume of coverage for these forms of news media.

Furthermore, few studies go beyond linguistic representations to extract underlying topics of COVID-19 in the media [5–7]. News discourses are susceptible to the cultural upbringing of its creators and conceived as a reflection of societal and cultural values [8, 9]. However, few studies cover how culture influence the volume of coverage provided by mass media. We contribute to the literature by examining the influence of culture values, during COVID-19 and pandemic variables (incidence and mortality) on the amount of news media attention given to COVID-19. Both culture and mass media are known to wield a strong influence in public health [10, 11]. Therefore, our work could lay the groundwork for policy makers to understand how culture and mass media potentially influence policies about pandemics.

Culture is multidimensional, consisting of values and behaviors transmitted between generations implying its enduring and corporate nature [12]. Constructs within culture can be independent, for instance not all cultural values are necessarily related to language [13]. Media sources like newspapers and news articles are addressed at a national level, and thus catered to the masses [9]. Therefore, cultural constructs that reflect group level sentiments are more appropriate for news media directed at the national level. Work by Hofstede produced a framework of cultural values at this level [14, 15]. In brief, power distance (PD) is the degree of perceived inequality of those form a lower stratum to those in authoritative positions. Individualism vs. collectivism refers to the strength of cohesion between individuals. Individualistic societies value independence, uniqueness, and personal freedom. Whereas collectivistic cultures value harmony, strong social cohesion, with clear demarcations between in-groups and out-group. Next, uncertainty avoidance (UA) is described as the degree of tolerance toward ambiguity in society. Cultures that rank high in femininity usually value modesty, cooperation, and high regard for overall quality of life. Antithetical to this, masculine cultures are more assertive, and stress competitiveness. Societies with high long-term-orientation (LTO) are ‘forward’ looking, pragmatic, perseverant, and value adaptive strategies. Indulgence vs. restraint was a latter addition, indulgent societies value free gratification of natural human desires, whereas restraint societies control behaviors by strict social norms.

The prevalence of news coverage can modulate the way health policies shape public sentiments [10, 11]. A systematic review found that wide news coverage of a previous H1N1 outbreak were disproportionate to the number of cases, leading to misperceptions and over-estimation of public health risks [16]. Evidently, fluctuations over public and governmental concerns about various affairs can stem from journalistic interests manifested by media outlets [11]. Mass media can boost or dampen government efforts to inform the public about existing policies to modify behavioral responses during disease outbreaks [10]. Either way, media coverage makes a substantial impact on public perceptions and subsequent behaviors [8]. Agenda theory explains that the impact of news and media may not tell us how to think about a particular topic, but rather what affairs are important, thus increasing the salience of targeted topics to the public. The quantity of coverage theory (QCT) extends this by asserting that it is the volume of coverage with repeated exposure, as opposed to the content of news media, that leads to higher saliency in the public’s mind [11]. These issues become the focal point in social discourses, naturally governments will react by implementing various policies accordingly [11].

Cultural values are also known to influence societal behaviors in response to policies about the pandemic [17]. A survey of 58 countries and found that higher UA evoked more risk avoidant behaviours, this relates to a lower degree of public gatherings which can curb infection rates [18]. Another study across several European countries concluded that countries with high PD, masculinity, and individualism have higher death ratios [19]. Countries that endorse individualism emphasize rights and freedom, and therefore less likely to adhere to restrictions [19]. Imposing harsher rules in high PD societies ironically reduce effectiveness, as these government may face higher opposition to lockdown enforcements [19]. Low masculine countries rely on strong social cohesion and consensus, an advantage to limiting the spread of COVID-19 [19]. Low LTO countries may react with hypervigilance, with short-term plans to stop the spread of the disease [19]. Cultural values therefore play a part in how people react during the pandemic, and the media respond to the rise and fall of cases and fatalities by generating coverage and articles accordingly. These studies suggests that cultural values are linked to the amount of attention the media gives to the pandemic, but there is virtually no empirical work to support this.

News media production and practices are not homogenous across the globe and subjected to socio-cultural influences. Cultural dimensions provide a context in explaining variation in news media across the world, and assist the relevance of news frames for a particular society [20]. For instance, journalists from individualistic and low PD cultures are likely to generate articles that cover social injustices and other inequalities in society [8]. Countries with higher LTO and UA are more likely to report on pragmatic solutions and facts rather than social inequalities [21]. One study evidenced that collectivistic cultures produced a wider diversity of COVID-19 topics in the news media [4], but whether such values can be applied to the quantity of coverage has not been explored. Consequently, this study contributes by examining how cultural values influence the amount of production of news media in the context of the COVID-19 pandemic.

Against this background, this study explores the extent to which cultural values, and pandemic variables (of incidence and mortality) predict the amount of media attention on the pandemic. Cultural values would also affect public behaviours during a pandemic. Naturally, infectious rates, fatalities, and imposed lockdowns would grab the media’s attention, resulting in large volumes of coverage. We hypothesis that cultural values would significantly predict global media attention toward COVID-19 across time after controlling for pandemic indicators such as incidence and mortality rates (Hypothesis 1). Next, the literature has evidenced that PD, LTO, individualism, and UA can influence media practices. We therefore hypothesis that these cultural values would significantly predict the amount of global media attention toward the pandemic (Hypothesis 2).

## Method

### Dataset

All media data were obtained from the News on the Web corpus of 12 billion words, gathered from over 7,000 online newspapers and magazines [22]. This dataset was created with funding from the National Science Foundation (NSF) and the National Endowment for the Humanities (NEH) to study contemporary language usage in countries where English is widely used. The corpus is updated with up to 200 million words extracted from over 300,000 articles per month. Sources of the articles are derived from six regions spanning 20 countries: North America (America, Canada), Oceania (Australia, New Zealand), Asia (Bangladesh, Hong Kong, India, Malaysia, Pakistan, Philippines, Singapore, Sri Lanka), Africa (Ghana, Kenya, Nigeria, South Africa, Tanzania), Europe (Ireland, United Kingdom), and the Caribbean (Jamaica). This largest global corpus of news media contains both local and global coverage, examples of prominent global networks include *ABC news*, *Fox news*, *CNN*, *BBC*, and *CNBC*. The corpus also amasses local news sources, for instance the UK is represented by a range of news outlets, such as the *Daily Mail*, *The Guardian*, *Leicester Mercury*, and *Manchester Evening News*.

### Media attention rate toward COVID-19

To track media attention toward COVID-19, we identified 10 target words—Coronavirus, Covid-19, Covid, nCoV, SARS-CoV-2, Wuhan Virus, Virus, Disease, Epidemic, Pandemic—and calculated the frequency of occurrence for all these target words from October 2019 through May 2020. While we acknowledge that terms like ‘Wuhan virus’ has been politicized, it was included to capture early articles in Dec 2019 to Feb 2020. We started data collection in October 2019 to establish a baseline before Covid-19 came into public consciousness and ended on May 2020 due to data availability at time of analysis. The combined prevalence of these 10 target words was tracked weekly from before the pandemic (Oct-Dec’19; to establish baseline prevalence for virus-related media coverage) to during the pandemic (Jan-May’20). Media Attention Rate was calculated per week, by country, where the total occurrence of these 10 keywords was the numerator and the total number of words in each country’s corpus was the denominator. This ratio was multiplied by 1 million to calculate the Media Attention Rate for each of the 18 countries. The Global Media Attention Rate was the combined scores of all 18 countries per week.

### COVID-19 variables

Pandemic variables were included to examine its relative importance toward media attention compared to cultural values. These were derived from the Oxford COVID-19 Government Response Tracker across 77 countries, providing daily and weekly updates of fatalities and confirmed cases [23]. *COVID-19 velocity* is the rate of increase of new cases presented as a percentage. This was conducted by having the number of new cases for the respective week in each country, divided by the total number of COVID-19 cases from the previous week, multiplied by 100. *COVID-19 prevalence* rate is the proportion of the population in a country infected with COVID-19. This was ascertained by having the cumulative number of COVID-19 cases for the respective week, divided by the respective country’s population, multiplied by 100,000. *COVID-19 mortality risk* is the rate of new deaths represented as a percentage. This was calculated by dividing new deaths for the respective week in the respective country by the total deaths from the previous week, multiplied by 100. *Cumulative mortality risk* is the proportion of deaths in each country’s population. The number of deaths for the respective week is divided by the respective country’s population, multiplied by 100,000.

### Hofstede’s cultural dimensions

We derived each country’s profile of Hofstede’s dimensions from the original cross-national surveys of IBM employees and consequent studies [24, 25]. The survey consists of 30 items, 24 of which pertains to the 6 cultural values. Individual responses to each question were first calculated at a national level. Answers on a five-point Likert scale were also averaged at a national level. Dichotomous items (Yes/No responses), or multiple-choice items were calculated based on a percentage of answers, or a combination of these responses. National level scores were weighted, and this provided a score for each dimension for each country ranging from 0 to 100.

### Analytical strategy

A study evidenced that the volume of online pandemic news peaked and grew exponentially between March ‘20 and May ‘20 for the news media dataset [26]. We therefore selected a time frame from Oct’19 to May ‘20 to ascertain a more varied coverage of COVID-19 news material. We then extracted cultural values of 18 equivalent countries from our news media dataset. Two countries (Jamaica and Kenya) were not present in the cultural dataset and were excluded from the analysis. As a result, our sample size consists of 539 data points, comprising of 18 countries over an 8-month period.

Mixed-effects regressions were conducted to model the impact of cultural values, pandemic severity, with Media Attention Toward COVID-19 as outcome. Intraclass correlations (ICC) were used to ascertain the degree of clustering in the data. Cultural values were included as fixed effects in the model, with time (weeks) as the random effect in the model. Predictor variables were entered progressively over two regression models to ascertain the change in its predictive value. All pandemic factors were entered into Model 1 (rate of new cases, deaths, etc.) and Model 2 included cultural variables. Multicollinearity was assessed with VIF tolerance scores with a conservative criterion of less than 5. All analyses were conducted on R v3.6.0.

## Results

### Descriptive statistics

**Fig 1:**
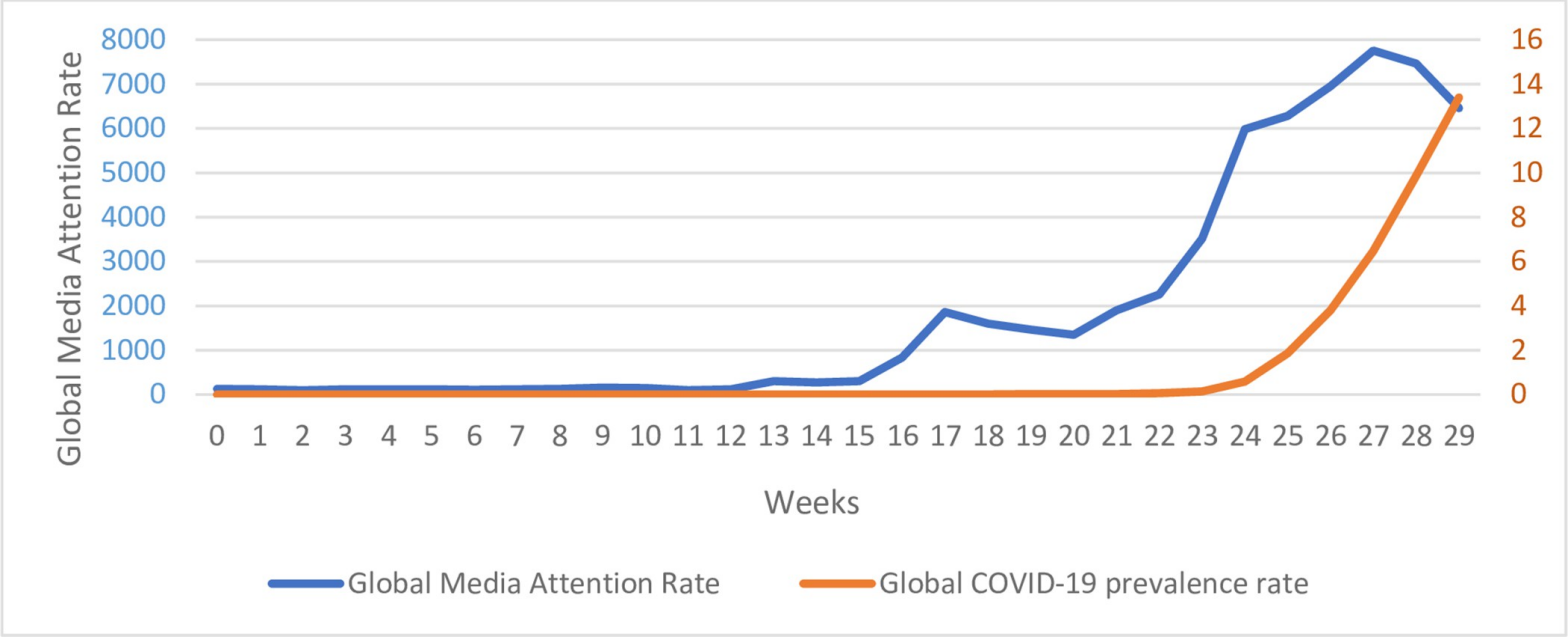
Global media attention rate and COVID-19 prevalence rate from a baseline period (Oct-Dec’19) to during the pandemic (Jan-May’20).

**Fig 2:**
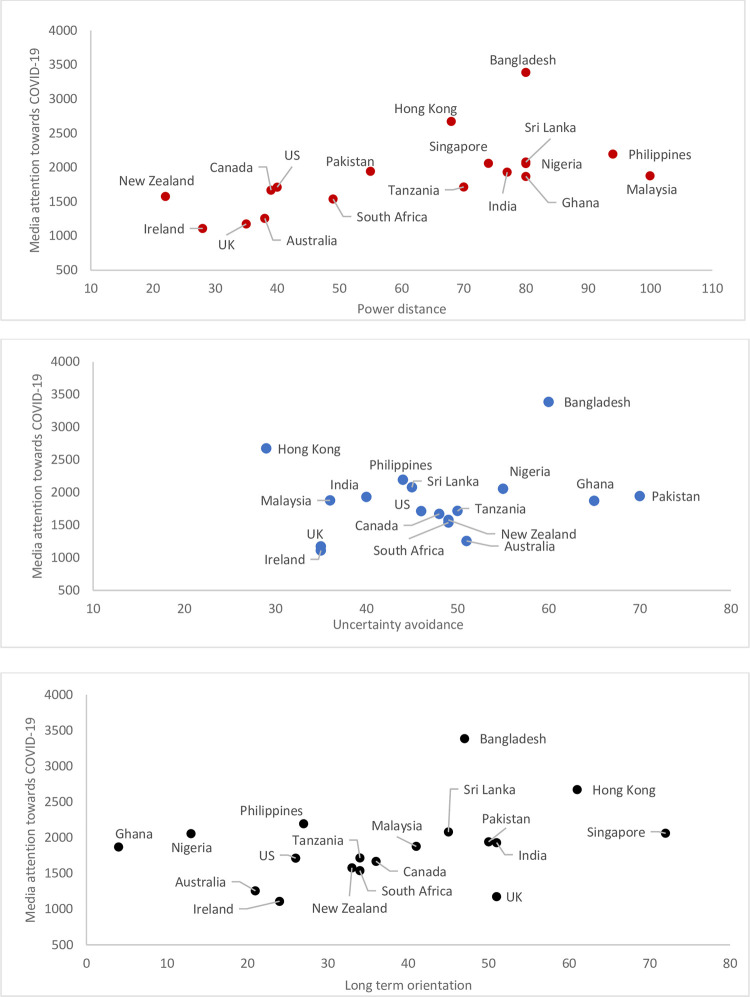
Scatterplots of power distance, uncertainty avoidance, and long-term-orientation against media attention towards COVID-19 for 18 countries.

Fig 1 presents media attention (as measured by the prevalence of all COVID-19 key words) from a baseline period of three months (Oct-Dec’19 to establish baseline prevalence for virus-related media coverage) to during the pandemic (Jan-May’20). Media attention experienced an exponential growth from a baseline of 100 words per million in October 2019 to 5500 words per million in April 2020—a 55-fold increase. The growth rate constant R_0_ (0.709±0.113) was significant, *p* < 0.01). The prevalence of global COVID-19 narratives dovetails the global Covid-19 incidence rates. Scatterplots revealed interesting relationships between cultural values and media diversity. Countries with higher long-term-orientation, Power distance, and Uncertainty Avoidance tend to have higher COVID-19 news media coverage (Fig 2).

### Predictors of global media attention toward COVID-19

Mixed-effects modeling were carried out in two successive blocks to ascertain the predictive value of pandemic variables and five cultural values (Table 1). ICC was 0.878 with the inclusion of the random intercept, indicating a high degree of clustering in the data. In the first model, only velocity (B = 19.7, *P* = .01) predicted global Media Attention toward COVID-19. The second model showed that velocity (B = 19.91, *P* = .003), mortality (B = 7.84, *P* < .001), power distance (B = 6.87, *P* = 0.017), uncertainty avoidance (B = 8.00, *P* = 0.027), and long-term orientation (B = 11.26, *P* < 0.01) emerged as significant predictors, controlling for covariates. There was no evidence for multicollinearity as the variance inflation factors (VIF) for all three models were below the conservative criteria of 5

**Table 1:** Mixed-effects regression models predicting media attention towards COVID-19.

	Model 1		Model 2	
	B(SE)	Sig.	B(SE)	Sig
COVID-19 Velocity ^a^	19.7(6.55)	0.01*	19.91(5.58)	0.003**
COVID-19 Prevalence Rate ^b^	-2365(1268.5)	0.25	-1757.28(766.15)	0.139
COVID-19 Mortality Rate ^c^	11.3(4.85)	0.07	7.84(2.06)	< .001**
Cumulative Mortality Risk ^d^	-3494.7(2470.2)	0.16	-3268.44(145.11)	0.175
Power Distance			6.87(2.87)	0.017**
Individualism			-4.74(2.50)	0.058
Masculinity			6.02(3.07)	0.050
Uncertainty Avoidance			8(3.60)	0.027*
Long-term Orientation			11.26(2.96)	< .001**

Note:

Sample consists of 539 data points.

^a^ Number of new COVID-19 cases for the respective week per country/Total COVID-19 cases in the previous week *100. Velocity is the rate of increase of new cases; represented as a percentage.

^b^ Cumulative number of COVID-19 Cases for the respective week/Respective Country Population x 100,000

^c^ Rate of increase of new COVID-19 deaths. New COVID-19 deaths for the respective week in the respective country/Total COVID-19 deaths in the previous week *100; represented as a percentage.

^d^ Cumulative COVID-19 mortality for the respective week/Respective Country Population x 100,000

*p < 0.05

** *p* < 0.01.

## Discussion

This is the first known study to model the impact of cultural values and pandemic severity on media attention across 18 countries. Our first hypothesis was supported, in which cultural values significantly predicted media attention, after adjusting for pandemic variables. This finding corroborates with the literature extant, where cultural values form an independent association with media diversity [4], and media consumption [27]. Our second hypothesis was partially supported, in which only PD, UA, and LTO were significant predictors of media attention.

Countries with higher Power Distance (PD) are linked to increased media attention toward COVID-19. It is noteworthy that individualism was not a significant predictor, given that PD and individualistic orientations together, are theoretically known to affect media practices [28, 29]. Countries with high PD and higher collectivism usually display lower press freedom [28, 29]—governments in these countries may exert greater control over media outlets. A recent study using the same data set found that only greater individualism associated with lower degree of COVID-19 media diversity [4]. Perhaps PD and individualism affect different aspects of media production. Countries with higher PD may want to exert greater control over society, which could encourage greater coverage of COVID-19 but not necessarily greater diversity of news.

Our data show that higher Uncertainty Avoidance (UA) is associated with increased media attention. Countries with high UA usually enforce a wider array of rules, with greater details regarding behaviors of individuals to mitigate the transmission of the disease (18). Subsequently, the media may respond by publishing more articles due to the quantity of governmental actions. Alternatively, countries high in UA generally display anxious predispositions about future events [14, 15], and a lack of information can amplify these fears [30]. In fact, intolerance toward ambiguity mediated the relationship between media exposure to COVID-19 and perceived stress [31]. Our findings corroborate with these explanations, where overwhelming media response to the pandemic could be attributed to low tolerance of uncertainty. The data also show that higher Long-term Orientation (LTO) is associated with higher media attention. Countries with higher LTO are likely to focus on future projections about the pandemic, which tend to be exponential, and this could result greater coverage in anticipation of escalating COVID-19 cases [19].

Our study contributes to the literature in two ways. First, our study contributes to the Quantity of Coverage Theory (QCT), with novel empirical evidence about the importance of cultural values in influencing the quantity of coverage. Our study also adds to the literature by quantifying the relationship between cultural values on news media production or news frames. This provides direct empirical evidence for theories that underly the intersection between culture and news media practices.

Second, on a practical front, our research provides insights to policy makers and communication outlets about the impact of culture on media control during the pandemic. During the onset of crises, it is possible for media outlets to be congruent with governmental actions creating a common narrative [32]. The media can therefore evoke certain national values to promote solidarity. For instance, societies with lower Uncertainty Avoidance tend to associate with higher pro-social behavior, and adopt better communicative strategies for vulnerable groups [10]. These countries could adopt policies that regulate the coverage of the pandemic to prevent dramatization of news. The media could direct public’s attention on important issues, or address ambiguity and uncertainty about the pandemic with the aim to increase social cohesion. Therefore, policy makers should consider how cultural values influence media coverage when dealing with public crises.

This study is not without limitations. Our main limitation is that our results may not generalize well to non-English speaking countries, the predictive value of cultural values may change as a function of different languages. Policy makers should consider that the relevance of cultural values on news media coverage might be mediated by language [33]. Next, our results are limited to cultural constructs set forth by Hofstede. Culture is multifaceted, future researchers may consider other measures of culture, such as those found in the GLOBE study [34]. We may not have full representation of different waves of the pandemic, and our results are limited to a particular time frame. Nonetheless, efforts were made to ensure sufficient volume of articles were produced based on past research.

Though the QCT does not dictate any standardized procedure to assess the volume of media coverage, we rationalized that an increase in the prevalence of keywords signifies and increase in the media’s attention toward COVID-19. Nonetheless, we encourage future research to investigate possible procedures [35–41] to validate our measures, within the QCT paradigm. Furthermore, the media’s attention toward COVID-19 may not equate to the public’s attention toward the pandemic. Future research could explore the efficacy of media attention to predict public attention [26, 42–46], and whether these discrepancies have any bearing on public sentiments [46–51] toward the pandemic. On the analytic front, future studies could consider OLS regressions with categorical variables assigned to respective countries, instead of the complexity of multiple level modeling.

## Conclusion

In conclusion, our study is one of the first to demonstrate the relative importance of cultural values regarding the amount of media attention given to the COVID-19 pandemic. Countries with *high* power distance, long-term orientation, and uncertainty avoidance are linked to increased media attention. Countries with higher uncertainty avoidance are likely to respond with hypervigilance to the current pandemic, and media outlets naturally follow by increasing coverage. Likewise, countries with greater long-term orientation may be concerned about future projections of the pandemic, which may garner media attention. In conjunction with other studies, findings for power distance suggests that different cultural values affect different aspects of media practices [52]. The study also contributes empirical evidence for the importance of culture in the QCT. Cultural values impact communicative practices and theories, especially when relating to media control and coverage. We hoped to have laid the groundwork for targeted public health communications that are culturally nuanced.
